# A prospective, open-label, non-comparative study of ambrisentan with anti-fibrotic agent combination therapy in the treatment of diffuse systemic sclerosis

**DOI:** 10.1186/s41927-018-0021-z

**Published:** 2018-05-15

**Authors:** Annemarie Schorpion, Max Shenin, Robin Neubauer, Chris T. Derk

**Affiliations:** 1 Hospital of the University of Pennsylvania, Philadelphia, USA; 2 Thomas Jefferson University Hospital, Philadelphia, USA; 3 Division of Rheumatology, University of Pennsylvania, 5th Floor White Building, 3400 Spruce Street, Philadelphia, PA 19107 USA

**Keywords:** Systemic sclerosis, Scleroderma, Ambrisentan, Combination therapy, Therapy, Mycophenolic mofetil, Treatment, Open label, Endothelin receptor blocker, Endothelin

## Abstract

**Background:**

Systemic Sclerosis is a multifactorial autoimmune rheumatic disease characterized by inflammation, fibrosis, immune dysregulation and vascular dysfunction.

**Methods:**

An open label, prospective, non-comparative study evaluating ambrisentan with an antifibrotic agent in diffuse cutaneous systemic sclerosis (dcSSc). Recruited 15 consecutive patients with dcSSc who were already on a stable dose of an antifibrotic agent and if they met inclusion criteria they were initiated on ambrisentan 5 mg/day for 12 months. Primary outcome measure was the modified Rodnan skin score (mRSS) while secondary measures were the short form 36 (SF-36) questionnaire, the Medsger severity score and pulmonary function studies.

**Results:**

Fifteen patients were recruited and ten patients completed all 12 months of the study. An intention to treat was used to analyze the data. There was statistical improvement of the mean mRSS and the perceived change in health component of the SF-36. The Medsger severity score and pulmonary function studies remained unchanged over the course of the study.

**Conclusion:**

Patients who tolerated the combination of an antifibrotic with ambrisentan had an improvement of their mRSS over the course of the study as well as an improvement of their perceived health.

**Trial registration:**

Clinicaltrials.gov, NCT01093885; March 2010.

## Background

Systemic Sclerosis (SSc) is an autoimmune rheumatic disorder of unknown etiology characterized by immune activation with autoantibody production, endothelial injury which leads to vascular dysfunction, and tissue fibrosis of both the cutaneous and visceral organs [1, 2]. It is believed that endothelial injury is the initial event which leads to vessel wall abnormality and an up-regulation of adhesion molecules that in turn attract inflammatory cells which transmigrate through the damaged vessel wall. Once these inflammatory cells migrate into the vessel wall they lead to local production of cytokines and growth factors. Tissue growth factor β (TGFβ) connective tissue growth factor (CTGF), interleukin (IL)-13 and IL-6 are just some of the cytokines and growth factors that are locally released in the vessel wall. On the other hand the injured endothelial cells release endothelin (ET)-1 and other vasoconstrictive chemokines. Platelets also play a significant role in the early steps of the pathogenesis of this disorder and they too are attracted to the injured endothelium and they in turn release platelet derived growth factor (PDGF), thrombin and thromboxane which in combination with the above mentioned growth factors and cytokines lead to fibroblast activation, with increased collagen synthesis and other extracellular matrix proteins that deposit in the vessel wall, and a subsequent transformation of fibroblasts into myofibroblasts. This results in functional and structural abnormalities of the vessel which in turn is theorized to cause similar changes to the organs that these vessels supply, such as the cutaneous tissue and the visceral organs [2–4]. Endothelin is one of the key pathogenic mediators in SSc having both a vascular and a fibrotic effect. There are three vasoactive peptides ET-1, ET-2 and ET-3 and from them ET-1 is the major isoform.

Endothelin-1 mediates its effects through two receptors, ETA, expressed on mesenchymal cells, and ETB, expressed on endothelial cells. In SSc ET-1 acts on fibroblasts, endothelial cells, smooth muscle cells, and macrophages. Endothelin-1 in SSc acts on the vascular smooth muscle cells to cause increased proliferation and altered vascular tone which in turn leads to vasoconstriction [5]. On endothelial cells it leads to increased proliferation and increased vascular permeability, while on fibroblasts it leads to increased cell proliferation, increased expression of α- smooth muscle actin, and transformation of fibroblasts to myofibroblasts with increased extracellular matrix deposition and decreased fibroblast apoptosis. Skin biopsies from SSc patients when stained with ET-1 reveal up-regulation in the microvessels and ET-1 levels in serum correlate with the extent of skin fibrosis [6]. In a study using bosentan, an ET-1 receptor blocker, 10 patients were treated in a prospective open-label, non-comparative trial for 24 weeks and both limited cutaneous systemic sclerosis (lcSSc) and dcSSc exhibited a statistical significant decrease in mRSS with a mean change in mRSS from baseline of 6.4 [7]. Randomized placebo controlled trials evaluating therapeutic agents for diffuse systemic sclerosis have been undertaken though often with negative results as these studies are often difficult to complete because of the rarity of the disease and the question if true placebo is an ethical option in such patients. Open label single center studies while inferior to the design of randomized controlled studies have in this particular disease helped establish certain agents as potential therapeutic agents in systemic sclerosis.

The hypothesis we based this study on is that endothelin receptor blockers in combination with disease modifying agents with antifibrotic properties will have additive influence on diminishing fibrosis, inhibiting cellular and humoral hyperactivity and interfering with smooth muscle proliferation in the vessel wall. Targeting the vasculopathic component of SSc and the potential overall disease modification effect this may have in the pathogenesis of the disease as a whole is an aspect that has as of yet not been clearly described in SSc but felt that it may influence disease progression based on the vasculopathic phenotype of the individual patient [8, 9] . The primary outcome measure of this study was to determine the benefit that ambrisentan in combination with an antifibrotic agent on the modified Rodnan skin score (mRSS) of early dcSSc patients.

Secondary outcome measures were to examine the effect of this combination therapy on the Medsger severity score [10] as well as quality of life measures as assessed by the SF-36 [11]. Safety parameters included clinical laboratory tests defining internal organ involvement, adverse events, physical examinations, vital signs and concomitant medications. Ambrisentan related adverse events such as anemia, fluid retention, peripheral edema, nasal congestion and flushing were of a primary focus.

## Methods

We designed an open label, single center study to determine the efficacy and safety of ambrisentan in combination with an antifibrotic agent (which could include mycopheolate mofetil, mycophenolic acid, methotrexate or cyclophosphamide). The patients would have had to have a diagnosis of dcSSc and onset of skin sclerosis less than 48 months from study entry and would have to already be treated with one of the above antifibrotic agents. All patients received ambrisentan 5 mg per day for a total of 12 months in combination with the antifibrotic agent. Once we received ethical approval from the institutional review board of the University of Pennsylvania we recruited consecutive patients both from within our own Rheumatology clinic but also from outside referrals from the local region. Patients had an initial baseline evaluation to determine if they met inclusion criteria. Once they were enrolled in the study they had monthly follow ups to capture safety issues and efficacy of the combination therapy. A full physical exam was performed at screen, 1,3,5,6,7,9,11 and 12 months and the SF-36 at screen, 1,3,6,9,12 months, though for our analysis we compared the screen to month 12 questionnaires. The Medsger severity score was evaluated at the screen visit and at 12 months, while the modified Rodnan skin score (mRSS) at screen, 6 and 12 months. The physical exam, Medsger severity score, mRSS were performed by the same investigator (CTD) to avoid inter-rater variability. Temporary dose reduction of ambrisentan below 5 mg/day was allowed for no more than 14 days in total during the study period. Monthly liver enzymes were followed during the study period and if aminotransferases were > 5 x the upper limit of normal (ULN) or a bilirubin of > 2 x ULN the study agent was stopped. If the aminotransferase level was > 3× ULN but <5xULN ambrisentan was stopped until the levels were < 3xULN. Pregnancy testing was performed every 3 months in all women capable of conception while two forms of contraception were recommend in all such patients.

The recruited patients had to be at least 18 years of age, fulfill the American Rheumatism Association criteria for systemic sclerosis, and satisfy subset criteria for diffuse cutaneous involvement as published by LeRoy et al. [12, 13] as the study inception was before the development of the 2013 ACR/EULAR SSc classification criteria [14]. Inclusion criteria included skin sclerosis ongoing for less than 48 months from study entry and a current regimen consisting of a stable dose of an antifibrotic agent. Previous history of using an alternate antifibrotic agent was permitted. Subjects were excluded if they had previous exposure to an endothelin receptor antagonist or were diagnosed with pulmonary arterial hypertension (PAH) and were on a PAH targeted therapy. If the total duration of anti-fibrotic treatment prior to study entry exceeded 48 months, or if they required corticosteroids equivalent to a dose of more than 20 mg/day of prednisone. Prohibited medications were biologic agents such as tumor necrosis factor (TNF)-alpha inhibitors within 6 months from study entry or rituxan within 12 months from study entry. Other exclusions included pregnancy or nursing, other scleroderma like illness, or other concomitant autoimmune rheumatic disorder, major surgery in the past 1 month, history of gastrointestinal hemorrhage within 6 months of initiation of study, unhealed peptic ulcers, congenital or acquired immune deficiency, history of severe viral illness, history of severe cardiovascular disease, congestive heart failure, liver dysfunction, current intravenous antibiotics. For the sample size calculation, we assumed a difference in the mean skin score from maximum to study end at 0.45, a value based on data from a previous study [15]. Using an α = 0.05, we calculated the sample size at *n* = 19 and we were able to recruit 15 patients into the study, all of whom agreed to the terms of participation and signed informed consent.

## Results

### Study population

**Fig. 1:**
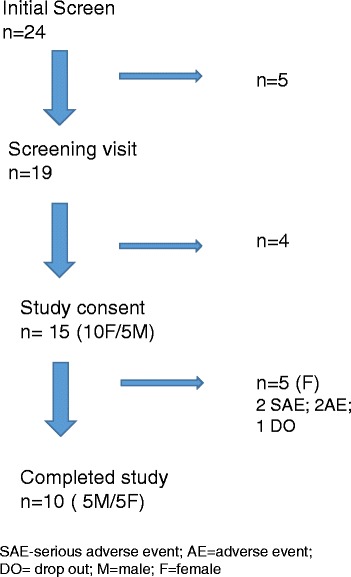
Study recruitment flowchart

A total of 15 patients were recruited for the study (Fig. 1), of which 10 were women and 5 were men. Fourteen of the patients were Caucasian and one was African American. The mean age was 47.6 ± 10.7 years old and 14 patients had a positive antinuclear antibody (ANA) with a median titer of 1:640. Ten patients had a speckled pattern and four a homogeneous pattern. One of our patients had a dual ANA pattern with both a speckled and a nucleolar pattern. The mean study entry white blood cell (WBC) count was 7907/μL blood, and mean erythrocyte sedimentation rate (ESR) 15.4 mm/h. At study entry, eight patients were taking a proton pump inhibitor, four a calcium channel blocker, five an angiotensin converting enzyme inhibitor, and four were taking daily aspirin. Six patients were taking daily prednisone (five patients were taking 5 mg per day and one was taking 10 mg per day) (Table 1).

**Table 1:** Study population characteristics (*n* = 15)

Age, mean (SD)	47.6 (10.7)
Race, n (%)	
White	14 (93)
Black or African American	1 (7)
Sex, n (%)	
female	10 (67)
male	5 (33)
Presence of ANA, n (%)	14 (93)
ANA Pattern^**a**^, n (%)	
speckled	10 (67)
homogeneous	4 (27)
nucleolar	1 (7)
Scl-70	4 (27)
RNP	0
SSA/SSB	0
dsDNA	0
WBC (per **μ**L), mean (SD)	7907 (2077)
ESR^**b**^, mean (SD), mm/h	15.4 (8.8)
Medication use, n (%)	
Antifibrotic agent^**c**^	
mycophenolate mofetil	12 (80)
mycophenolic acid	2 (13)
methotrexate	1 (7)
cyclophosphamide	1 (7)
prednisone	
(none)	9 (60)
5 mg/d	3 (33)
10 mg/d	1 (7)
PPI	8 (53)
CCB	4 (27)
ACEi	5 (33)
ARB	0
statin	2 (13)
ASA	5 (34)
NSAID	1 (7)
Clinical symptoms, n (%)	
ILD	4 (27)
SIBO	1 (7)
GERD	9 (60)
Hypothyroidism	2 (13)
Raynaud’s	15 (100)
DU	4 (27)
GAVE	1 (7)
Esophageal dysmotility	5 (33)
Pericardial effusion	1 (7)
SRC	1 (7)
HTN	1 (7)
Arthritis	2 (13)
Sicca	1 (7)
Calcinosis	1 (7)
Intestinal Pseudobstruction	1 (7)
Disease duration months(SD)	
Raynaud’s	37 ± 40
Skin sclerosis	19 ± 15

*ANA* anti-nuclear antibody, *ESR* erythrocyte sedimentation rate, *PPI* proton pump inhibitor, *CCB* calcium channel blocker, *ACEi* angiotensin-converting enzyme inhibitor, *ARB* angiotensin receptor blocker, *NSAID* non-steroidal anti-inflammatory drug, *ILD* interstitial lung disease, *SIBO* small intestinal bacterial overgrowth, *GERD* gastroesophageal reflux disease, *DU* digital ulcer, *GAVE* gastric antral vascular ectasia, *SRC* scleroderma renal crisis, *HTN* hypertension

^a^1 patient had both a speckled and nucleolar pattern; 1 patient did not have the presence of an ANA detected; 1 patient’s ANA status was unknown

^b^2 patients had unknown levels of ESR at study entry

^c^1 patient received both cyclophosphamide and mycophenolate mofetil

The antifibrotic agents used included 12 patients on mycophenolate mofetil, two on mycophenolic acid and one on methotrexate. One of the patients on mycophenolate mofetil had also received cyclophosphamide prior to the initiation of the current antifibrotic agent. Five patients terminated the study early, two because of a serious adverse event (SAE) that required a hospitalization, two patients had an adverse event (AE) that caused them to terminate the study, while one patient was lost to follow-up. One patient had worsening anemia from gastric antral vascular ectasia (GAVE) at 6 months into the study and had to be hospitalized, one patient had anxiety, migraine headache, and adynamic ileus at 6 months which also required hospitalization. Another patient had lower extremity swelling at 1 month and one patient had pruritus, and tingling of the extremities and trunk also at 1 month. Finally, one patient was lost to follow up after the 6 month visit and on subsequent follow up communication the patient was well with no adverse events and did not follow up as he did not want to continue in the study.

### mRSS

**Fig. 2:**
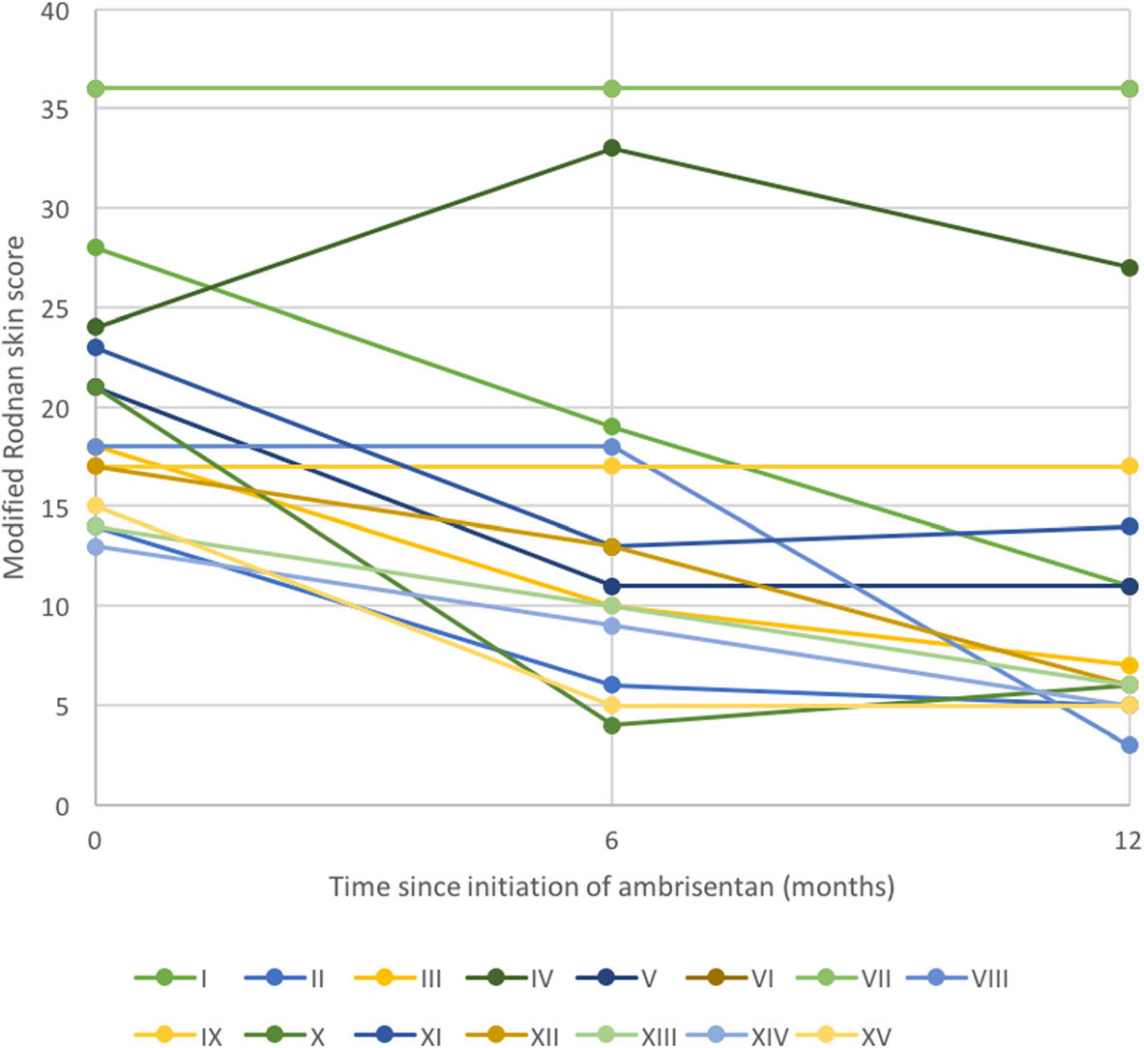
Individual patient modified Rodnan skin scores at 0, 6 and 12 months of the study

The primary outcome measure of the study was the mRSS. We used an intention-to-treat analysis for all patients who entered into the study and for missing data on patients who terminated the study early we imputed values based on the last observation carried forward. For the five patients who terminated early, in 2 the imputed values were collected at 1 month into the study and for 3 patients at 6 months into the study. The mean mRSS at study entry was 21 (standard deviation (SD) 7.4, range 13–36). At 6 months, the mean mRSS was 16 (SD 10.8) and at 12 months, 13 (SD 11.2) (Fig. 2). Using one-way ANOVA analysis, comparing the mean mRSS at the time of study entry and study end, we observed a mean difference in mRSS of − 8 (*t* = − 5.08, *p* = 0.000167).

### Medsger severity score

The degree of visceral organ involvement was quantified using the Medsger severity score. The Medsger severity score includes nine categories (general, peripheral vascular, skin, joints, muscular, gastrointestinal, pulmonary, cardiac and renal) representing areas of potential organ involvement by SSc. Each category is rated on a scale from 0 to 4, with higher numbers signifying more severe organ disease. At study entry, the median Medsger severity score for the population studied (*n* = 15) was as follows: peripheral vascular 1 and skin 2. Median scores for all other categories were 0. At study end, the median peripheral vascular score was 1 and the median skin score was 1. Median scores for all other categories remained 0.

### Pulmonary function studies

**Fig. 3:**
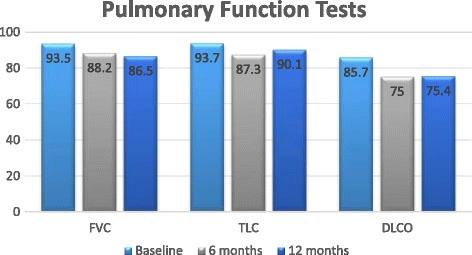
Pulmonary Function Tests (PFTs) at 0, 6 and 12 months since initiation of ambrisentan

All study patients were asked to have complete pulmonary function tests (PFT’s) performed at study entry, 6 months and at the end of the study at 12 months. For the purpose of our study, we compared mean values for forced vital capacity (FVC), total lung capacity (TLC) and pulmonary diffusing capacity (DL_CO_) for all patients (*n* = 12) at all three time points (Fig. 3). The mean FVC was 93.5% predicted at study entry, 88.2% predicted at 6 months, and 86.5% predicted at 12 months. The TLC was 93.7% predicted at study entry, 87.3% predicted at 6 months, and 90.1% predicted at 12 months. The DL_CO_ was 85.7% predicted at study entry, 75% predicted at 6 months and 75.4% predicted at 1 2 months. Using a one-way ANOVA to analyze the data, there was no statistically significant change observed. All of our patients had echocardiograms at study entry and all patients had normal ejection fractions and no suggestions of elevated right ventricular systolic pressure, as such patients were excluded.

### SF-36

Every 3 months each patient was asked to fill out the SF-36 questionnaire. For this study, we compared the results at study entry with those at study end of the patients who completed all 12 months of therapy (*n* = 10). The SF-36 is a 36 item questionnaire which describes quality of life across eight different domains. Physical functioning; role limitations due to physical health; role limitations due to emotional problems; energy/fatigue; emotional wellbeing; social functioning; general health; and perceived change in health. The mean SF-36 score at study entry was 62 (23.3) and 65.9 (25.7) at study end, a difference that was not statistically significant (*p* = 0.092). However, the mean perceived change in health component at study entry was 37.5 (39.5) compared to 55 (38.7) at study end, a significant difference of 17.5 (*p* = 0.025). No statistically significant differences were detected among scores in the other 7 categories of the SF-36 questionnaire.

## Discussion

Systemic Sclerosis continues to lack a universally acceptable therapy though consensus statements have recommended such agents as methotrexate and mycophenolate mofetil as acceptable therapies for dcSSc patients and this is based on open label studies, cases series and randomized controlled treatment trials [16]. Data from the two well designed randomized controlled trials for SSc related interstitial lung disease, the Scleroderma lung trial 1 and 2 have emphasized the statistical significant results on FVC in the group taking cyclophosphamide in the first study and the equivalent results of mycophenolate mofetil to cyclophosphamide in the second trial. While the absolute FVC changes are small, both studies also have suggested that these agents have significant skin results based on the mRSS [17, 18]. With the current study we attempt to look at the potential of combination therapy in dcSSc, targeting fibrosis, inflammation and the vasculopathy of this disease. Using a baseline antifibrotic agent such as methotrexate, mycophenolate mofetil or mycophenolic acid in combination with an ERA such as ambrisentan which may have a vascular modifying effect in SSc patients we attempted to see the tolerability of this regimen and its effect on the mRSS and other disease parameters and more specifically the SF-36 and Medger severity score. With the combination therapy in early dcSSc patients we attempted targeting fibrosis, inflammation and the vasculopathy of this disease. Patients who tolerated ambrisentan in combination with an antifibrotic agent showed an overall improvement in their mRSS and their perceived physical health, while over the 12 months of the study they did not have progression of their disease severity based on the Medsger severity scale. While the study has all of the limitations expected from an open label non-comparative study it does reveal that patients who tolerated the regimen had stability in their disease process and had improvement in their skin scores. Our inability to recruit the correct amount of patients to our study based on our power calculations may have also negatively affected some of our results where we observed some difference but not quiet statistically significant such as the total SF-36 score at study entry vs study end. Recruitment was hampered because many patients newly diagnosed with dcSSc at our institution are placed on antifibrotic agents as well as PAH related vasodilatory therapy either for PAH or Raynaud’s. Another limitation is the fact that 5 patients did not complete the whole study based on SAEs, AEs and a single drop out. While the SAE’s and AE’s may have been related to the study agent patients who tolerated the study agent fared well as per our results and this can help future studies looking at this combination therapy to design based on such an estimated drop out from SAEs and AEs While our results don’t clearly define if the improvement in the mRSS is based on delayed immunomodulatory effects from the antifibrotic agents the patients were already on at study entry, the natural course of the disease or specifically the combination therapy what we can conclude is that patients who tolerated the combination therapy and who had early dcSSc disease remained stable over the 12 months of the study among all of the Medsger severity domains and had an improvement in their skin scores and perceived changes in health. For this to be looked in more detail a randomized placebo controlled comparative study needs to be undertaken to decipher how much of this effect relates to the antifibrotic agent itself and how much from the combination of an antifibrotic and an ERA, and in addition to measures used in this study tissue and serum based biomarkers may also help us to better identify the potential of combination therapy for this disorder. Also more detailed measures of the individual organ involvement beyond the Medsger severity score will also give a better definition of organ involvement and treatment related changes. In the current study biomarkers were not explored as the inception of the study was previous to their more well defined description and use in clinical research [19]. While ERA’s are only approved for pulmonary arterial hypertension in the US, bosentan has received approval in the EU for the reduction of the number of new digital ulcerations in SSc patients. As systemic sclerosis is a multifactorial illness characterized by early inflammation, immune dysregulation, vascular dysfunction and fibrosis, targeting just one of these components of the illness may not be a realistic way at looking at treating this autoimmune rheumatic disorder. Targeting all components that take part in the development of this disease may be a more realistic approach.

## Conclusion

Combination therapy with an antifibrotic and an endothelin receptor antagonist in patients with dcSSc improved the mRSS and the patient’s perceived physical health over a 12 month treatment period while disease severity did not worsen during the treatment period. A large randomized study is needed to further evaluate the utility of combination therapy in dcSSc.

## Abbreviations

AE: Adverse event
ANA: Antinuclear antibody
CTGF: Connective tissue growth factor
dcSSc: Diffuse cutaneous systemic sclerosis
DLCO: Diffusing lung capacity
ESR: Erythrocyte sedimentation rate
ET: Endothelin
FVC: Forced vital capacity
GAVE: Gastric antral vascular ectasia
IL: Interleukin
lcSSc: Limited cutaneous systemic sclerosis
mRSS: Modified Rodnan skin score
PAH: Pulmonary arterial hypertension
PDGF: Platelet derived growth factor
PFT: Pulmonary function test
SAE: Serious adverse events
SD: Standard deviation
SF-36: Short form-36
SSc: Systemic sclerosis
TGF-β: Tissue growth factor beta
TLC: Total lung capacity
TNF: Tumor necrosis factor
ULN: Upper limit of normal
WBC: White blood cell
